# Cleaning the Flue in Wood-Burning Stoves Is a Key Factor in Reducing Household Air Pollution

**DOI:** 10.3390/toxics10100615

**Published:** 2022-10-17

**Authors:** Mizanur Rahman, Hans Petersen, Hammad Irshad, Congjian Liu, Jacob McDonald, Akshay Sood, Paula M. Meek, Yohannes Tesfaigzi

**Affiliations:** 1 Pulmonary and Critical Care Medicine, Department of Medicine, Brigham and Women’s Hospital, Harvard Medical School, Boston, MA 02115, USA; 2 Chronic Obstructive Pulmonary Disease Program, Lovelace Biomedical Research Institute, Albuquerque, NM 87108, USA; 3 Applied Sciences, Lovelace Biomedical Research Institute, Albuquerque, NM 87108, USA; 4 Department of Internal Medicine, University of New Mexico School of Medicine and Miners Colfax Medical Center, Raton, NM 87740, USA; 5 College of Nursing, University of Utah, Salt Lake City, UT 84102, USA

**Keywords:** wood stove, wood smoke, household air pollution, stove age, particulate matter, stove maintenance

## Abstract

In experimental settings, replacing old wood stoves with new wood stoves results in reduced personal exposure to household air pollution. We tested this assumption by measuring PM_2.5_ and levoglucosan concentrations inside homes and correlated them with wood stove age. Methods: Thirty homes in the Albuquerque, NM area were monitored over a seven-day period using in-home particulate monitors placed in a common living area during the winter months. Real-time aerosol monitoring was performed, and filter samples were analyzed gravimetrically to calculate PM_2.5_ concentrations and chemically to determine concentrations of levoglucosan. A linear regression model with backward stepwise elimination was performed to determine the factors that would predict household air pollution measures. Results: In this sample, 73.3% of the households used wood as their primary source of heating, and 60% burned daily or almost daily. The mean burn time over the test week was 50 ± 38 h, and only one household burned wood 24/day (168 h). The average PM_2.5_ concentration (standard deviation) for the 30 homes during the seven-day period was 34.6 µg/m^3^ (41.3 µg/m^3^), and median (min, max) values were 15.5 µg/m^3^ (7.3 µg/m^3^, 193 µg/m^3^). Average PM_2.5_ concentrations in 30 homes ranged from 0–15 μg/m^3^ to >100 μg/m^3^. Maximum PM_2.5_ concentrations ranged from 100–200 μg/m^3^ to >3000 μg/m^3^. The levoglucosan levels showed a linear correlation with the total PM_2.5_ collected by the filters (R^2^ = 0.92). However, neither mean nor peak PM_2.5_ nor levoglucosan levels were correlated with the age (10.85 ± 8.54 years) of the wood stove (R^2^ ≤ 0.07, *p* > 0.23). The final adjusted linear regression model showed that average PM_2.5_ was associated with reports of cleaning the flue with a beta estimate of 35.56 (3.47–67.65) and R^2^ = 0.16 (*p* = 0.04). Discussion: Cleaning the flue and not the wood stove age was associated with household air pollution indices. Education on wood stove maintenance and safe burning practices may be more important in reducing household air pollution than the purchase of new stoves.

## 1. Introduction

Exposure to wood smoke (WS) is increasing not only in low-income countries but also in America, Canada, Europe, the Taigas in Canada, Alaska, and Siberia [1]. Climate change and other factors contribute to the rise in prevalence of wildfire events [2], causing populations in large areas being exposed to outdoor air pollution and toxic particulate matter (PM). In addition, household air pollution is a major concern [3,4] because approximately one-third of the world’s population, comprising over 2.4 billion people, still uses solid fuels, such as wood, coal, or biomass (vegetable remains and dung), for cooking and heating their homes [5,6]. During the winter months, 30% of ambient fine particles (PM_2.5_) mass stems from wood burning used for heating and cooking in some areas of the United States (US) [7]. More recently, exposure to household air pollution has increased during the COVID-19 pandemic as people were confined to their homes for longer periods [8].

According to the guidelines published by the World Health Organization (WHO) in September 2021, the levels of 24 h mean particulate matter (PM) concentration is 45 μg/m^3^ for PM_10_ and 15 μg/m^3^ for PM_2.5_ [9]. However, indoor PM_2.5_ concentrations in the US often exceed health-based air quality standards, especially in homes that use stoves for cooking or heating. In Montana, mean indoor PM_2.5_ concentrations of 45 µg/m^3^ and 51 µg/m^3^ were reported in homes with wood stoves [10,11], exceeding the WHO 24 h standard for PM_2.5_ of 15 µg/m^3^. However, in addition to PM, levoglucosan content is a large fraction of the emitted fine particles from wood burning [12]. Levoglucosan is a product of pyrolysis generated during the combustion of wood and a major constituent of PM_2.5_, and, therefore, it has been proposed as a tracer of WS [12].

An estimated 3.8 million premature deaths are caused each year from illnesses attributable to household air pollution due to heating or cooking with inefficient stoves using either solid fuel or kerosene [5]. Exposure to household pollutants is particularly high among women and young children, and this contributes to many deaths in children under 5 years of age. These deaths are primarily due to acute lower respiratory infections such as pneumonia and in adult women due to chronic obstructive pulmonary disease (COPD) [13,14]. However, even exposure to low levels of WS PM can cause oxidative stress in lung cells and elicit airway inflammation. Thus, exposure to WS can be a major cause of respiratory illness in all susceptible individuals [15] and has been implicated in respiratory illness, including COPD exacerbations [16], lower respiratory infections [17], and cough and wheezing [18,19]. Epidemiological studies suggest that WS exposure may cause an increased risk of infection and reduced lung function [20,21,22]. Several controlled exposure studies also demonstrated a clear association between exposure to WS particulates and respiratory dysfunction [23,24]. A 25 µg/m^3^ increase in 6-d mean indoor PM_2.5_ concentrations was associated with the presence of lower respiratory tract infection in children [25]. Exposure to WS affects not only the respiratory system but also increases the risk for cancer (lung, head and neck, cervical), interstitial lung disease, cardiovascular diseases, hypertension, low birth weight, and reduces growth rate of children [26,27].

Over 90% of the total PM from biomass burning is smaller than 2.5 μm, which can enter the alveolar region and pass into circulation [28,29]. Because wood stoves are major sources of household air pollution, several intervention strategies have been implemented to reduce indoor PM_2.5_. Open fires generate high concentrations of WS particulate matter of 2000–30,000 µg/m^3^, and the use of improved wood stoves reduces exposures to the 1000–5000 µg/m^3^ range [11]. Modern technologies in biomass combustion, such as automatic small-scale wood pellet appliances and larger domestic heating plants, are commonly more efficient and emit much lower levels of PM [30]. Changeout programs of older wood stove models with new EPA-certified wood stoves in Libby, Montana, showed a >70% reduction in indoor PM_2.5_ concentrations [10,31]. These studies determined a drop of basal mean values from 51.2 to 15 µg/m^3^ PM_2.5_ after the changeout of stoves [10]. The follow-up study showed that the mean PM_2.5_ level of 45.0 µg/m^3^ before changeout was reduced to 21.0 µg/m^3^ over the following three winters. However, over subsequent winters, the average concentrations across homes varied, and several homes actually showed increased concentrations [11]. Despite the overall reduction in indoor pollutants, the study suggested that not only the introduction of a new wood stove but other factors contribute to the level of pollutants. Recently, the same research group proposed that a lack of cleaning of chimneys may also contribute to indoor pollution [32]. Therefore, simply replacing old woodstoves with newer improved ones may not have a long-term benefit of reducing exposure to WS PM_2.5_.

Additional studies are necessary to determine whether newer wood stoves reduce household air pollution [33]. Confirmatory studies should not only have accurate measurements of PM emission from wood stoves but also consider including chemical markers that define the source of pollutants. Therefore, the main objective of the present study was to document the age of wood stoves, in addition to important factors, such as stove maintenance and frequency of cleaning the flue, as these variables were not included in earlier studies. Further, earlier investigations did not consider the possibility that other indoor pollutants, such as PM_2.5_, generated from cigarette smoking can affect indoor air quality. The present study measured the wood pyrolysis product levoglucosan to confirm that the PM_2.5_ stems from wood burning specifically. Therefore, by including both PM_2.5_ and levoglucosan, the present study was designed to elucidate whether the age of wood stoves is a key determinant of reducing household air pollution in “real-life settings” or whether other factors, such as flue cleaning, affect indoor air pollutant levels.

## 2. Materials and Methods

### 2.1. Study Population

The sample for this investigation was drawn from those currently enrolled in the Lovelace Smokers Cohort (LSC), who reported yes to the question, “have you been exposed to WS over the last year”. Further details of the LSC have been described previously [34,35,36]. Most LSC participants were recruited through newspaper or television advertisements, and ongoing recruitment continues using these methods in Albuquerque, an urban, diverse, high-altitude Southwestern community.

### 2.2. Study Design

This analysis was part of a larger study that tested the development of a self-report questionnaire concerning exposure to household WS. Exposure to WSwas self-reported in response to a question administered at study entry as part of the general health survey. The question “Have you ever been exposed to WS for 12 months or longer” provided no additional details about the type, intensity, and duration of WS exposure. The original research design was a cross-sectional sample, monitoring the particulate matter and levoglucosan concentrations over seven days in 30 homes. The homes (*n* = 30) were selected from those originally contacted by a study coordinator and those contacted by word of mouth. All individuals were enrolled during the heating season when wood stoves were active in the home to obtain real-world experience. This analysis reports on these 30 homes’ internal environment exposures.

Demographic information such as age, gender, ethnicity, race, smoking history, and history of respiratory disease was obtained using the American Thoracic Society (ATS)-DLD-78 questionnaire, with some questions added about in-home exposures to smoking. Demographic information concerning wood stove maintenance and burning details were also asked, including what type of fuel was burned and cleaning of the flue and stove. The thirty homes were monitored over the seven-day period using in-home particulate monitors placed in a common living area during the winter months of 2013–2014. The Teflon filter was conditioned a minimum of 24 h prior to and after sample collection at 25 °C and 40% relative humidity. Filter samples were collected with a Personal Environmental Monitor that had a 2.5-micron size selective inlet (PEM, Model 200, PEM-10-2.5, MSP Corporation, Shoreview, MN, USA) (Figure S1). Real-time aerosol monitoring was performed by using a DustTrak Aerosol Monitor (Model 8520, TSI, Inc., Shoreview, MN, USA). The aerosol sample enters through a multi-nozzle, single-stage impactor to remove particles with an aerodynamic diameter (AD) larger than 2.5-µm in diameter. Particles smaller than the impactor cut-point were collected on a 37 mm diameter filter. Two different types of filters were used during this study. For the first 12 deployments, PTFE Zefluor filters, pore size, 3.0-μm (Part No. 60230, Pall Life Sciences, Ann Arbor, MI, USA) were used. However, it was observed that due to heavy particulate loading in some residences and a long sampling time (1 week), the pressure dropped across the filters and reduced the sampling flow rate or caused the failure of the pump. Due to this consequence, Zefluor filters were replaced with PallFlex Membrane Filters (Type: Fiberfilm T60A20, Pall Life Sciences, Ann Arbor, MI, USA) for the remaining household deployments. With this change, no drops in the sampling flow rate were observed despite the high loading of the filters.

### 2.3. In-Home Particulate Samples and Analysis

PM_2.5_ Filter Sampling System: Dust Track was set up in the room with the wood stoves, and PM was measured. The measurement of PM in Dust Track identified WS as the major constituent of the filter particulate loading. The PM_2.5_ filter sampling system consisted of two major parts: the PEM and the Leland Legacy Air Sampling Pump. The PEM (Model 200, PEM-10-2.5, MSP Corporation, Shoreview, MN, USA) is a lightweight personal sampler for collecting airborne particles in the PM_10_ and PM_2.5_ size range with a cut-point of 2.5 μm AD, as used in this study (Figure S2). The flow rate through the sample was maintained at 10 ± 1 L/min to maintain a 2.5 μm cut-point. The Leland Legacy Air Sampling Pump (SKC, Inc., Eighty-Four, PA, USA) was used to provide the required sampling flow rate through the PEM. The PEM sampler was installed on a vertical rod about 4–5 ft. from the ground (typical breathing zone while sitting), with the inlet holes aligned parallel with the floor to avoid gravitational settling. Additional detail on the sampling system can be found in the Supplementary Materials.

Sampling Deployment Procedure: Pre-deployment began with the Personal Environmental Monitor (PEM) being prepared by cleaning the impaction surface, and a thin film of grease was applied to the impaction surface to minimize particle bounce and re-entrainment. The filter was weighed using a microbalance (Model MX5, Mettler Toledo, Columbus, OH, USA) and installed in the PEM. Then, the PEM was connected to the sampling pump, and the sampling flow rate was adjusted to achieve a 10 ± 1 L/min flow rate. The flow rate was measured by installing a TSI flow meter (Model: 4100, TSI, Inc., Shoreview, MN, USA) between the sampling pump and the PEM. Pickup and post-deployment began with both samplers (DustTrak and PEM) being stopped with the final flow rate, and the pressure drop across the PEM sampler was measured and recorded similarly to pre-deployment. The filter was weighed and stored in a −80 °C freezer until levoglucosan analysis could be performed. Additional detail on the sampling deployment procedure is described in the Supplementary Figure S2.

Chemical Analysis: Levoglucosan (1, 6-anhydro-b-D-glucopyranose), a cellulose combustion product, is a tracer species for WS, mainly because of its high resistance to degradation. Levoglucosan levels were determined in the collected PM to determine whether the PM was primarily from wood burning, as 23% of the study participants were also current cigarette smokers. The amount of levoglucosan analysis of the filter extraction solution was determined by GC-MS. The analysis was performed by Desert Research Institute, Reno, Nevada, USA, using the following analytical method. Details of the chemical analyses are described in Supplementary Materials. Due to storage and chemical analysis failure, levoglucosan values were obtained in only 23 of 30 homes. Data were reported in ng/m^3^ units.

Statistical Analysis: Summary demographic statistics for continuous variables consisted of means and standard deviation (S.D.), and categorical variables are presented as proportions. We conducted Pearson’s correlations to examine associations of the woodstove age with the average of PM_2.5_, peak value of PM_2.5_, or levoglucosan levels of in-home particulate measures (*n* = 30) and whether or not the stove was maintained regularly (yes/no) or the flue cleaned (yes/no). Based on our hypothesis of associations with the subject’s proximity to the stove (based on three questions: (a) Over the past week, when wood was burning in the stove/fireplace, there was some smoke in the room? (b) When wood is burning, how close to the stove/fireplace are you? (c) Usually, when wood was burning in the stove/fireplace, I was in the same room?), stove age, number of cigarettes smoked per day, cleaning the flue, cleaning the stove, income, and education, we performed a linear regression model with backward stepwise elimination to determine the factors that would predict household air pollution measures. All analyses were conducted using SAS version 9.4 (Cary, NC, USA).

## 3. Results

### 3.1. Demographic Characteristics of Subjects and the Homes

The mean age of the study participants was almost 60 years, 43% were male, and about half were Hispanic (Table 1). Approximately a quarter (23%) were current smokers, and two-thirds (66.7%) of the individuals reported some chronic conditions (Table 1). The majority of the study participants reported living in a home with 5 rooms and 1–2 individuals residing in the home. The stove was reported to be serviced in the last year in 65.5% of the households, with the flue being cleaned in over half of the sample within the past year. Only 30% reported using a humidifier, and only one household used an air filter in their home.

**Figure 1:**
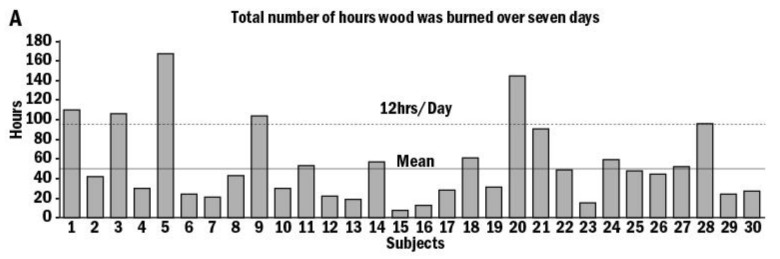

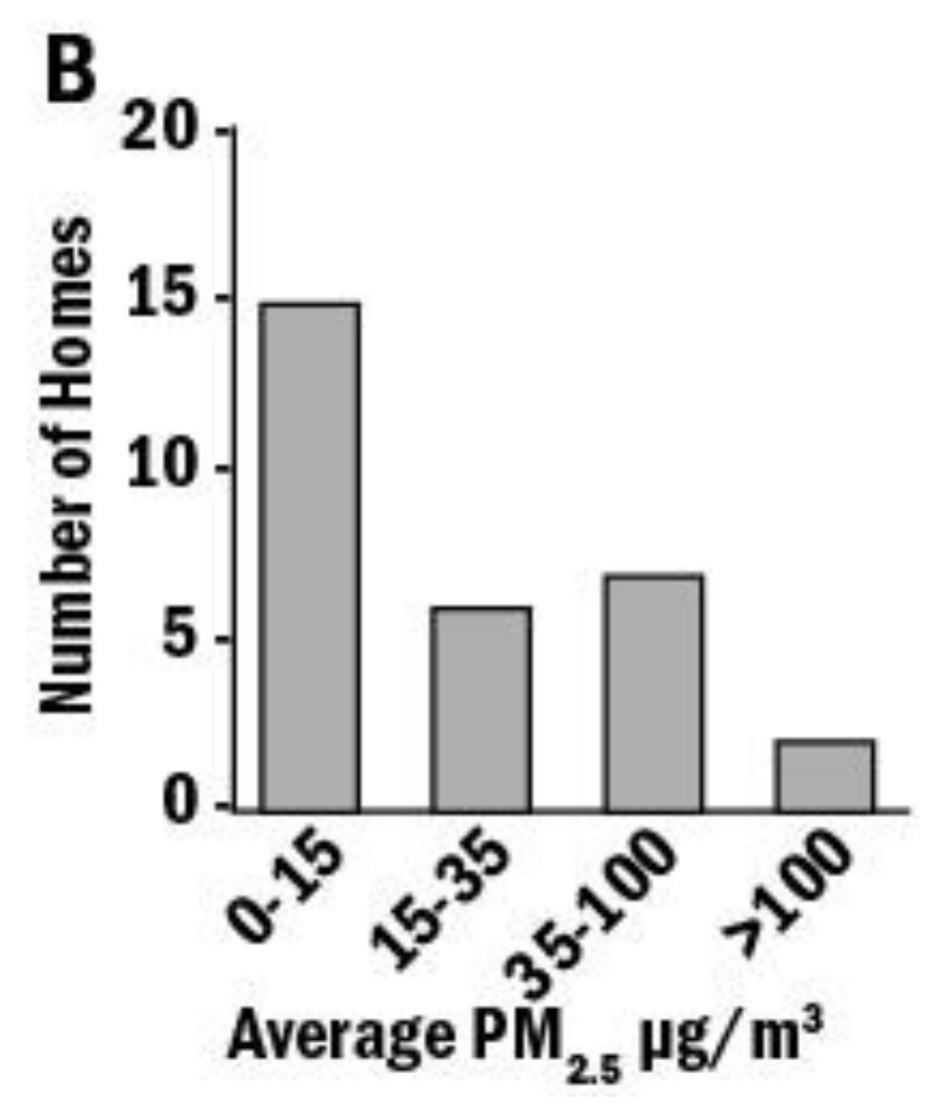
Total number of hours of wood burning in 30 homes. Total wood-burning time over the 7 days was calculated, and the mean wood-burning time over the test week was 50 ± 38 h with only one home burring 24 h a day (**A**). Average PM_2.5_ frequency in different homes. Particulate mass was collected on filter, and total PM_2.5_ emission was recorded over the test week from the 30 homes. Average PM_2.5_ range was >100 μg/m^3^ in 1 home, 0–15 μg/m3 in 15 homes, 15–35 μg/m^3^ in 6 homes, and 35–100 μg/m^3^ in 8 homes (**B**).

**Figure 2:**
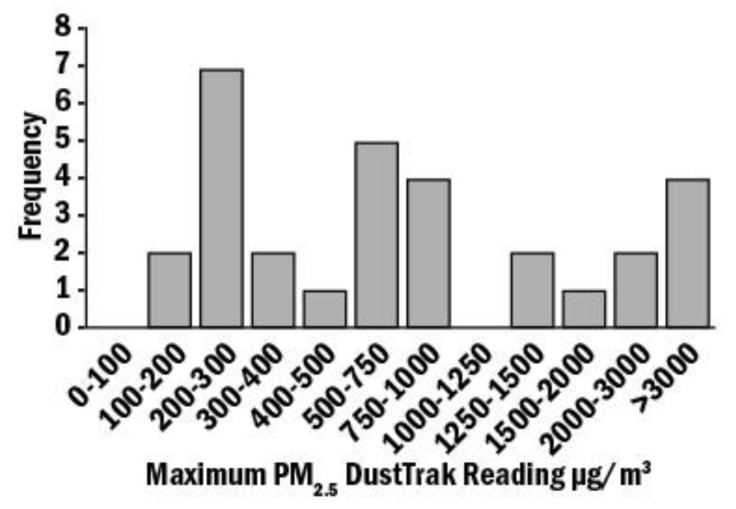
PM_2.5_ concentration in DustTrack from 30 homes. A DustTrak Aerosol Monitor was used to monitor real time aerosol. Mean PM_2.5_ ranged from 100–200 μg/m^3^ to >3000 μg/m^3^ in the 30 homes.

Among the 30 households in this study, 73.3% used wood as their primary source of heating, and 60% burned wood daily or almost daily. The mean burn time over the test week was 50 ± 38 h, with only one home burning 24 h a day (Figure 1A). On average, the measured PM_2.5_ ranged between 0–15 μg/m^3^ and >100 μg/m^3^ (Figure 1B). Over the 6 days, the average PM_2.5_ was >100 μg/m^3^ in only 1 home, 15 homes (50% of the sample) showed 0–15, 6 homes (20%) measured 15–35 μg/m^3^, and in 8 homes, PM_2.5_ ranged between 35 and 100 μg/m^3^ (Figure 1B). Interestingly, the peak measurements ranged from 100–200 μg/m^3^ to >3000 μg/m^3^ in four homes (Figure 2).

### 3.2. WS Was the Cause of the Measured PM 

**Figure 3:**
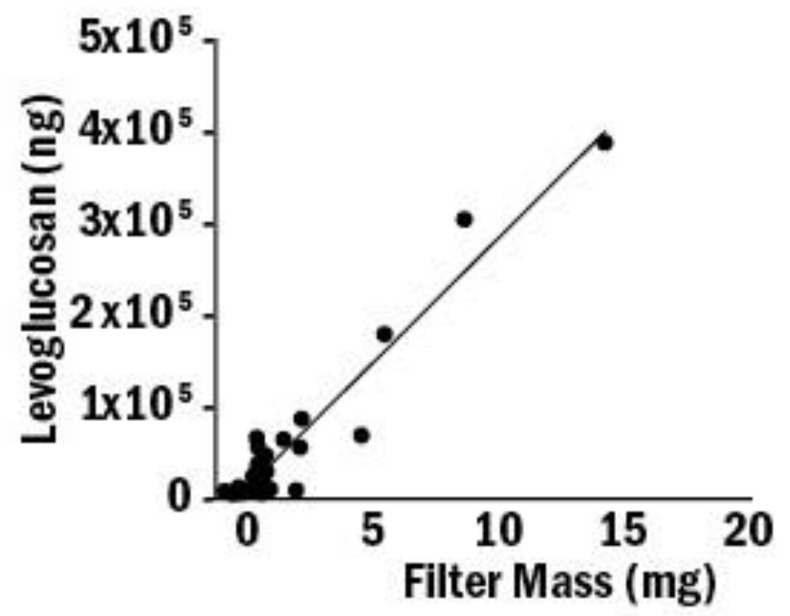
Linear association between levoglucosan and filter mass. Levoglucosan was extracted from the filter, and the amount of levoglucosan was determined by GC-MS analysis from the filter extraction solution. The level of levoglucosan was associated with filter mass, R^2^ = 0.92, *p* =< 0.01.

There was a linear relationship (R^2^ = 0.83) between PM_2.5_ in the filter measurement concentration and DustTrak average concentration reading. Levoglucosan collected by filters showed a positive linear relationship with (R^2^ = 0.92, *p* < 0.01) the particulate mass collected on the filter (Figure 3).

### 3.3. Age of Wood Stoves Was Not Associated with the PM and Levoglucosan

**Figure 4:**
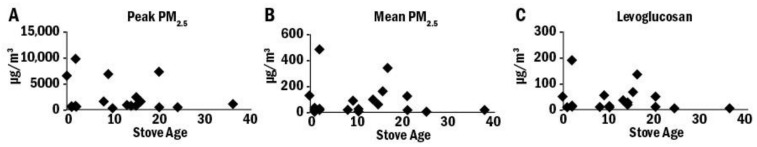
Age of the wood stoves was not correlated with PM_2.5_ or levoglucosan. Neither Peak (**A**) nor mean (**B**) of PM_2.5_ emission was associated with the age of the wood stoves. Similarly, the level of levoglucosan was not associated with the age of the stoves (**C**).

The result was shown as Figure 4.

### 3.4. The Age of Wood Stoves Did Not Play a Role in PM_2.5_ or Levoglucosan Levels 

The age of wood stoves was not correlated with either PM_2.5_ or levoglucosan (Figure 4). Over the seven-day measurement period, peak PM_2.5_ emission (R^2^ = 0.01, *p* = 0.555) (Figure 4A) or mean PM_2.5_ values (R^2^ = 0.00, *p* = 0.893) (Figure 4B) were not associated with the age of the stoves. Similarly, the mean concentration of the levoglucosan (R^2^ = 0.07, *p* = 0.230) was not associated with the age of the wood stoves (Figure 4C).

### 3.5. Cleaning of Wood Stoves Was Associated with PM_2.5_ Emission

Using a univariate linear regression model, we identified that only the variable “flue cleaning” was associated with PM emission (Table 2). When using the full model that includes all variables in multivariate linear regression, we did not find an association of any of the co-variates with PM (Table 3). After backward selection, only stove flue was left in the model, so the final model is the same as the result from the univariable table. The linear regression model showed that the average PM_2.5_ was associated with reports of cleaning the flue with a beta estimate for stove flume of 35.56 (3.47–67.65) and R^2^ = 0.16 (*p* = 0.03).

## 4. Discussion

The current study identified that the age of wood stoves is not correlated with PM emission, but it is rather the maintenance and cleaning of the flue that is correlated with household air pollution due to wood stoves. Furthermore, the PM_2.5_ emission level was positively associated with the level of levoglucosan. In the current study, the level of indoor PM_2.5_ was significantly higher than the WHO’s recommended levels, suggesting that people who use wood stoves are exposed to high levels of WS, even in high-income countries. Our findings are similar to the levels of indoor PM_2.5_ in homes with wood stoves that were measured in earlier studies [10,11,31,37,38]. Over 24 h, PM_2.5_ concentrations ranged from 24 to 60 µg/m^3^, whereas sampling over a 2 h cooking period exceeded 1000 µg/m^3^ [38]. The peak levels of PM emission are expected to be high during the cooking or heating periods. In another study, the measured PM_2.5_ levels were higher (1910 to 6030 µg/m^3^) than in our study when the measurements were taken during the cooking period [39]. However, these high concentrations in PM emissions may be the result of the types of stoves used. Mean PM_2.5_ levels of 5310 µg/m^3^ and maximum PM_2.5_ levels of 13,800 µg/m^3^ were measured in the homes that used open fire and in some homes with Plancha or Lorena wood stoves. However, all the homes in our study used stoves with chimneys, and the PM_2.5_ was measured over the 7-day period. The peak value of PM_2.5_ in our study exceeded 3000 µg/m^3^ during the cooking and heating period in four homes (>10% of homes studied). These levels of PM_2.5_ concentrations are usually thought to be present only in low-income countries [40].

Changing older wood stoves with newer EPA-certified wood stoves was widely encouraged to help reduce household air pollution [10,31,37,41]. The wood stove changeout program in Libby reduced indoor PM_2.5_ concentrations by >70%, but the first study did not provide any information on the sustainability of the effect. The follow-up study conducted multiple samplings over subsequent winters and found large variability in the average PM_2.5_ levels across the homes: several homes had higher concentrations than the concentration pre-changeout [11]. In the original study [10], only one measurement was taken following the changeout. Although multiple samplings from 21 homes in the follow-up study suggested, on average, a 53% reduction in PM_2.5_ in 16 homes, 7 homes demonstrated no reduction post-changeout. Interestingly, samplings from seven of the homes exhibited even higher levels of PM_2.5_ than pre-changeout [10]. Another study revealed [41] that the wood stove changeout program reduced phenolics and PAH compounds on average by 64%, while the mass of PM_2.5_ was reduced by only 20%. However, in that study, EPA-certified stoves were installed with efficient burning woods. The efficient combustion of wood in modern, certified stoves may also contribute to lowering PM emissions. Wood with higher moisture content produces a higher quantity of PM compared to dry wood [42,43]. High moisture content causes incomplete wood combustion resulting in high emission of PM. Therefore, indoor WS levels are likely determined by the type of wood used, moisture content, the combustion appliances, as well as the combustion phase, which affects PM generation [44,45,46]. These studies determined that types of wood and airflow play a role in the amount and different types of PM, including the generation of pyrolysis products. Studies with more controlled burning conditions are needed to determine the contribution of these factors to PM generation by wood burning.

Next, we measured levoglucosan, which forms from the pyrolysis of starch and cellulose of wood. Hydrolysis or biodegradation, even the combustion of fossil fuel, does not produce levoglucosan. Cellulose combustion generates levoglucosan, which is considered a tracer for biomass burning [47,48,49,50]. Depending on the air supply, the relative range of levoglucosan to total particle emission from wood burning was reported to be 3–17%. Although, as a fine particle, levoglucosan constitutes a large fraction of total emitted particles from wood burning. Studies with levoglucosan measurements as a marker of wood smoke from wood stoves are sparse. The measurements of levoglucosan in our study were carried out over 7 days, and levoglucosan is stable over 10 days [51]. The levoglucosan level detected in the homes of the current study was similar to the levels previously reported (mean 300 ng/m^3^) in a different study [52]. The PM_2.5_ level was associated with levoglucosan, but no association was found between levoglucosan and the age of the stoves, which is in line with the finding on the association between PM_2.5_ and the age of the stoves. However, reports of cleaning the flue were able to explain 16% of the variance in the PM_2.5_ level [32]. In addition to best burning practices, operating and maintenance, including flue cleaning, may have a significant influence on the emission of PM. A previous study [32] showed higher PM_2.5_ in the homes that reported cleaning their chimney more than 12 months before the sampling period compared to those that cleaned chimneys within 6 months of the sampling period. Regular cleaning of the flue is crucial to reduce indoor PM emissions, as the ashes can accumulate and clog the passage of the WS outdoors. Further, air intake vents can improve wood-burning efficiency [47]. Cleaning of the flue may increase wood-burning efficiency by air intakes and generate less PM, thus reducing PM emissions. To reduce indoor household pollution, education on wood stove maintenance and safe burning practices are more important than replacing old stoves with new stoves.

The limitations of this study include the relatively small number of homes studied, and extensive characterization of the WS in all 30 homes could provide detailed information on exposures experienced by all study participants. Future studies should inquire about the experience and skill of the individual who operates and maintains the wood stove and include homes without wood stoves for comparison. Furthermore, determining the effect of different types of wood in smoke generation and identifying the wood type that may be less likely to clog the flue would be the most efficient path to reduce indoor PM emissions. Although all the homes were in the same area, the effect of ambient air pollution on the indoor air should be considered. Further, 23% of the household participants currently smoked, and although smoking did not seem to be a factor associated with PM, it is possible that it could have affected the measurement. The strengths of our studies are the positive association of PM_2.5_ with levoglucosan levels, confirming that the PM indeed represents WS.

## 5. Comparative Section

In line with earlier studies (45–58), our study further determined that levoglucosan is a useful wood-burning marker to study indoor air pollution in connection with WS. Furthermore, the homes included in this study were selected at random, but all the homes were with similar architecture and ventilation, and the type of wood stoves are representative of the stoves used in the area. Indoor air pollution varies in different geographical regions or areas. We are not aware of an earlier study that was conducted in New Mexico that investigated the age of wood stoves and household air pollution.

## 6. Conclusions and Future Perspectives 

Although changeout with EPA-certified woodstoves was suggested as a strategy to minimize exposure to PM_2.5_, our study suggests that the age of wood stoves is not associated with the level of indoor PM but rather with flue cleaning. In addition, better characterization of PM_2.5_ is recommended to ensure the origin is warranted. Additionally, further studies with bigger sample sizes are needed to elucidate whether moisture content in the wood and cleaning the flue improve indoor air quality. Installation of proper stoves, operation following best burning practices with proper wood selection with maintenance, and regularly cleaning flue may reduce the level of PM emissions from wood stoves. Whether smoke from different types of wood or moisture content may result in the rapid clogging of the flue should also be investigated. Finally, whether flue cleaning will reduce household air pollution to an extent that translates into health benefits will need further investigation.

## Figures and Tables

**Table 1:** Characteristics of the study participants in 30 homes studied.

Variable	Mean/Number	Percentage/SD
Age (<46 years)	3	10.0%
Gender Male	13	43.3%
Hispanic	14	46.7%
Reported Chronic Illness	20	66.7%
Currently Smoking	7	23.3%
Education (>high school)	27	90.0%
Annual Household Income (expense less than income)	3	10.0%
Number of individuals in the home	2.29	0.87
Number of rooms in the home	4.87	0.51
Stove maintained in last year (%Yes)	19	65.5%
Flue cleaned in the last year (%Yes)	16	53.3%
Used a humidifier (%Yes)	9	30%
Used an air filter (%Yes)	1	3.3%

**Table 2:** Univariate linear regression.

Variable	Beta-Est.	95% CI	*p*-Value
Income	8.416	−8.7–25.6	0.35
Education	0.8414	−27.6–29.2	0.95
Stove_Clean	9.3082	−27.5–446.1	0.62
Stove_flue	35.5638	3.1–68.0	0.04
Cig.per.Day	−0.6049	−3.9–2.6	0.72
Proximity	0.6271	−15.5–16.8	0.94
Stove Age	−0.2954	−2.4–1.8	0.78

**Table 3:** Multivariate linear regression.

	Est.	95% CI	*p*-Value
Intercept	11.22	−196.1–173.7	0.91
Income	7.02	−17.1–31.2	0.58
Education	4.15	−35.9–44.3	0.84
Stove_Clean	2.7	−39.8–45.2	0.9
Stove_flue	32.84	−9.3–75.0	0.15
Cig.per.Day	0.44	−3.6–4.5	0.83
Proximity	−0.47	−17.7–16.8-	0.577
Stove Age	0.02	−2.2–2.3	0.987

## Data Availability

Not applicable.

## Supplementary Materials

The following supporting information can be downloaded at: https://www.mdpi.com/article/10.3390/toxics10100615/s1, Figure S1: PEM-10-2.5 in disassembled form. Multiple jets of single stage impactor can be seen on the red inlet part. The impaction surface on the left shows collected particles greater than 2.5-μm. The center part shows the filter (mostly black with a white boundary).; Figure S2: PEM installed on a rod attached to an electric cooler enclosure
