# Supplementary material for: Cleaning the Flue in Wood-Burning Stoves Is a Key Factor in Reducing Household Air Pollution
Source: Toxics. 2022 Oct 17;10(10):615. doi: 10.3390/toxics10100615 (PMC9609584; doi:10.3390/toxics10100615)
Supplement: Supplementary file 1 [file toxics-10-00615-s001.zip › toxics-1921032-supplementary.pdf]

toxics

Supplementary Materials: Cleaning the Flue in Wood-Burning Stoves Is a Key Factor in Reducing Household Air Pollution

Mizanur Rahman, Hans Petersen, Hammad Irshad, Congjian Liu, Jacob McDonald, Akshay Sood, Paula M. Meek and Yohannes Tesfaigzi

Materials and Methods

In-home Particulate Samples and Analysis

The pump is a battery-operated personal sampling pump, and it can operate in 5-15 L/min flow range. Since the battery of these pumps cannot last a whole week, the pumps were operated by using a 110 VAC household power outlet. The PEM (**Figure S1**) and the Leland Legacy pump were connected by 3/8″ Polyflo tubing. The sampling pump does make a continuous noise while operating. This noise can be more than 60dB while operating at high flow rates and with associated high pressure drops. Since the sampling systems were to be placed in subjects' living areas with furnace/woodstove, it was decided to sound-proof the sampling system. During initial tests, it was observed that the temperature of the enclosure (used for soundproofing) with the sampling pump could exceed the maximum allowable operating temperature of the pump. To solve this high temperature problem, the sampling pump was placed in a Koolatran Compact Cooler (**Figure S2**) (Model P20, Koolatran, Corp., Brantford, Canada). These are electric coolers designed for use in minivans and SUVs with 12-V power supply. AC adapters were used to operate these with 110 VAC household power outlets. Holes were drilled in the electric cooler to run sampling tube from the sampling pump to the PEM. The PEM sampler was installed on a vertical rod about 4-5 ft. from the ground (typical breathing zone while sitting), with the inlet holes aligned parallel with the floor to avoid gravitational settling. Five sampling systems with PEM, and Koolatran cooler were prepared for simultaneous sampling in up to five houses.

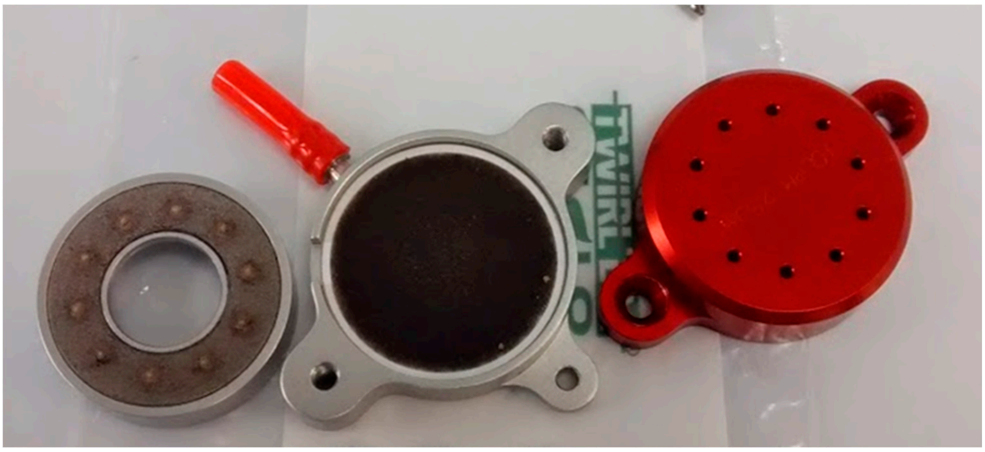

Figure S1. PEM-10-2.5 in disassembled form. Multiple jets of single stage impactor can be seen on the red inlet part. The impaction surface on the left shows collected particles greater than 2.5- m. The center part shows the filter (mostly black with a white boundary).

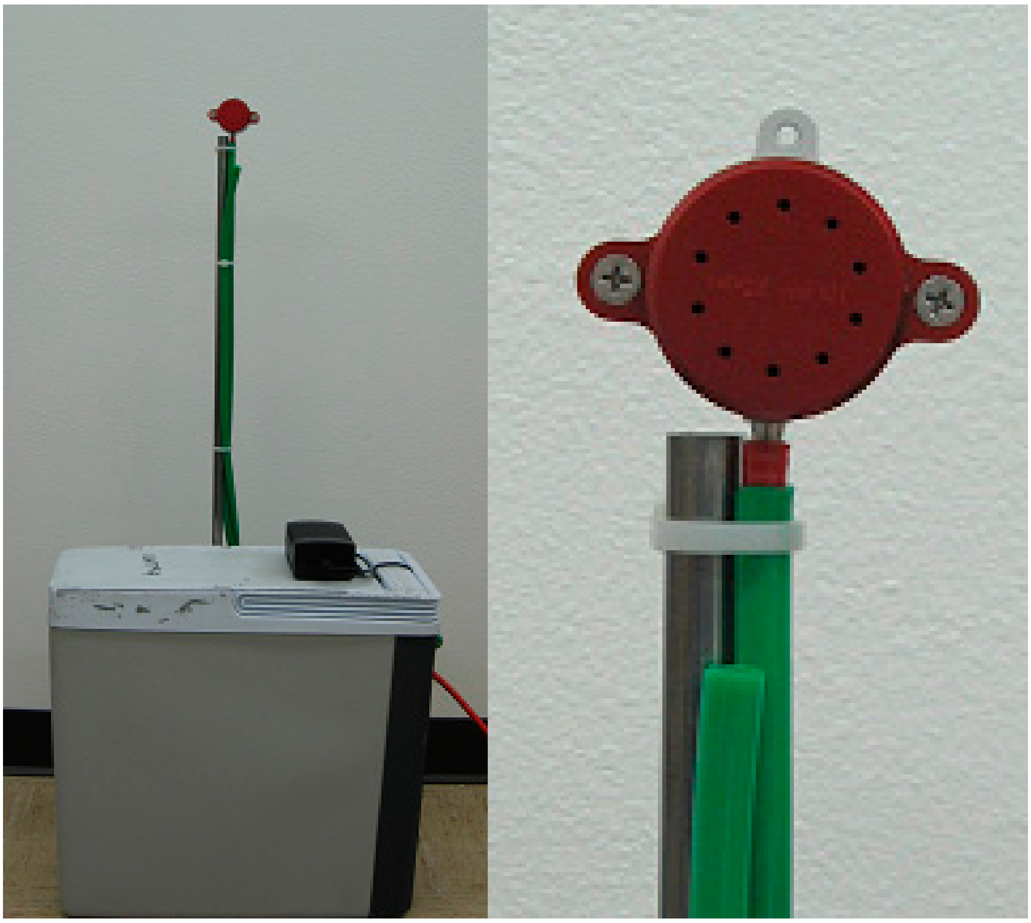

Figure S2. PEM installed on a rod attached to an electric cooler enclosure.

Sampling Deployment Procedure

Corresponding pressure drop across the sampler was also recorded. Deployment in the home required the sampler being placed in the room with the furnace/wood stove. The electric cooler and sampling pump were connected to a power outlet. After starting the pump, the top cover of cooler was closed and sealed using duct tape. The distance of the sampling system from the furnace/wood stove was measured and recorded, and an approximate sketch of the room was prepared to show the relative location of the stove and the wood stove/furnace.

Chemical Analysis

Samples were extracted using Accelerated Solvent Extractor (Dionex ASE 300). Prior to extraction, deuterated internal standard (Levoglucosan-d7, Cambridge Isotope Laboratories, Inc., Tewksbury, MA, USA) was added to each sample. Filters (37mm) were extracted twice with HPLC grade acetone. Each extraction was done for 15 minutes at 1500 psi and 80°C. Two extracts of each sample were then combined and concentrated by rotary evaporation at 20°C under vacuum to ~1ml and filtered through a 25mm PTFE Whatman 0.22μm filter. Samples were transferred to a deactivated 1.5ml clear glass vial with a cap and PTFE/silicone septum. Extracts were evaporated to a small volume (near dryness) under moisture filtered UHP nitrogen and resuspended in acetonitrile. Extracts then underwent trimethylsilyl derivatization, utilizing a mixture of bis-(trimethylsilyl)-trifluoroacetamide (BSTFA) + 1% (wt) trimethylchlorosilane with pyridine (silylation grade) to enhance detection sensitivity.

Samples were analyzed using Varian 4000 GC/MS system equipped with a CP-8400 autosampler. A 1 μL injection onto 30m 5% phenylmethyl silicone fused-silica capillary

column (DB-5MS+DG, J&W Scientific, Folsom, CA) was performed. Compounds and corresponding deuterated internal standards were quantified by selective ion monitoring. Individual neat standards were purchased from Sigma-Aldrich Co. and used to make calibration solutions. A five-level calibration was performed for each compound of interest, and the calibration check (using a median calibration solution of standards) was run every ten samples to check for the accuracy of the analysis. If the relative accuracy of measurement (defined as a percentage difference from the standard value) was less than 20%, the instrument was recalibrated, and samples were reanalyzed.
